# Three-dimensional measurement and analysis of Mandibular Molar Distalization assisted by micro-implant anchorage combined with clear aligner

**DOI:** 10.12669/pjms.40.3.7759

**Published:** 2024

**Authors:** Gengbing Lin, Mengwei Chen, Nan Guo, Xie Shi

**Affiliations:** 1 Gengbing Lin, Department of Stomatology, Fujian Provincial People’s Hospital, Affiliated People’s Hospital of Fujian University of Traditional Chinese Medicine, Fuzhou 350004, Fujian, China; 2 Mengwei Chen, Department of Orthodontics, Fujian Key Laboratory of Oral Diseases & Fujian Provincial Engineering Research Center of Oral Biomaterial & Stomatological Key lab of Fujian College and University, School and Hospital of Stomatology, Fujian Medical University & Institute of Stomatology, Fujian Medical University, Fuzhou 350004, Fujian, China; 3 Nan Guo, Department of Stomatology, Fujian Provincial People’s Hospital, Affiliated People’s Hospital of Fujian University of Traditional Chinese Medicine, Fuzhou 350004, Fujian, China; 4 Xie Shi, Department of Orthodontics, Fujian Key Laboratory of Oral Diseases & Fujian Provincial Engineering Research Center of Oral Biomaterial & Stomatological Key lab of Fujian College and University, School and Hospital of Stomatology, Fujian Medical University & Institute of Stomatology, Fujian Medical University, Fuzhou 350004, Fujian, China

**Keywords:** Clear aligner, Mandibular molar distalization, Three-dimensional measurement, Micro-implant anchorage

## Abstract

**Objective:**

To investigate the effect of micro-implant anchorage combined with a clear aligner on the efficiency of mandibular molar distalization and the protection of anterior teeth anchorage, provide reference for clinical scheme design.

**Methods:**

This is a prospective study. Seventeen patients who were treated in the Orthodontics Department of the Hospital of Stomatology affiliated to Fujian Medical University from 2019 to 2021 and used Invisalign clear aligners to move mandibular molars distally were included and divided into two groups according to anchorage types: Group-A and Group-B. Group-A (ten cases) were treated without micro-implant anchorage, while Group-B (seven cases) were treated with micro-implant anchorage nails for enhanced anchorage. The effect of micro-implant anchorage on crown and root distal movement of mandibular molars and the difference in three-dimensional movement between mandibular molars and mandibular central incisors were analyzed.

**Results:**

The crown distalization efficiency of mandibular first and second molars in Group-B was 68.66% and 71.02%, respectively, which were higher than those in Group-A(p<0.05). The mandibular central incisors in Group-A showed labial displacement and a small amount of elongation, while those in Group-B showed less anchorage loss(p<0.05). In Group-A, the crown was tilted in the distal direction and moved in the buccal direction during mandibular molar distalization(p<0.05). While in Group-B, the crown was tilted in the distal directio (p<0.05) and the mandibular second molar was depressed(p<0.05).

**Conclusion:**

In the process of mandibular molar distalization assisted by micro-implant anchorage combined with a clear aligner, better protects the anchorage of the mandibular central incisor and improves the efficiency of the molar crown distalization.

## INTRODUCTION

The beauty and health of teeth has always been a topic of concern.^1^,^2^ Recent years have witnessed a spurt of progress in digital technology, and clear aligners are increasingly favored by doctors and patients.^3^-^5^ It has been shown in related studies that a clear aligner is beneficial to distal mandibular molars without losing the anchorage of the anterior teeth.^6^ However, the limited resolution of two-dimensional images and overlapping bilateral images make the possibility of errors in their measurements unavoidable.^7^ There may be changes in jaw position before and after treatment. It may not be able to reflect the position change of mandibular molars with respect to the mandible by setting measurement coordinates or overlapping only based on the maxillary structure. In this study, by combining CBCT with digital models, the characteristics of mandibular molar distalization with a clear aligner were analyzed, and the effects of micro-implant anchorage on molar distalization efficiency and anterior teeth anchorage protection were compared, thus providing a reference for clinical design.

## METHODS

This is a prospective study. Seventeen patients who were treated in the Orthodontics Department of the Hospital of Stomatology affiliated to Fujian Medical University from 2019 to 2021 and used Invisalign clear aligners to move mandibular molars distally were included as subjects. All of them were divided into two groups according to anchorage types, of which Group-A (ten cases) had no additional anchorage, while Group-B (seven cases) used micro-implant anchorage to assist in mandibular molar distalization. Patients in Group-B were implanted with anchorage nails in the bilateral external oblique area before the distalization of the mandibular first molar, and the anchorage nails were pulled with the precise cutting place of the ipsilateral canine area and the lingual buckle on the buccal side of the mandibular second premolar. All patients had their molars distally displaced using V-partten, digital models of their molars were made in STL before treatment (TO) and when the mandibular molars were in place (T1), and CBCT was photographed simultaneously. Subsequently, the digital models in STL of the initial bit and target bit (TX) were derived from ClinCheck software.

### Ethical Approval:

The study was approved by the Institutional Ethics Committee of Fujian Medical University & Institute of Stomatology, Fujian Medical University on December 9, 2021 (No.: [2020]87), and written informed consent was obtained from all participants.

Combination and overlapping of crown and root models of mandible and teeth: the digital model of the patient’s mandible in the T0 stage was imported into Geomagic wrap 2017, and the clinical crown was preserved by “Surface Clipping”. CBCT was imported into Mimics Medical 20.0 in the T0 phase, and the STL model of mandibular teeth was reconstructed by a “Split Mask” to distinguish mandibular teeth from surrounding tissues. In Geomagic wrap 2017, the crowns of the above two models were overlapped by “N-point alignment + optimal fitting”. After overlapping, the two were spliced by cutting, “curvature bridging” and “curvature internal hole filling”, and marker points were constructed at the mid-point of the mandibular molar face, the mid-point of the apical area and the mid-point of the incisor margin and apical point, forming a dental crown-root integration model. With the same method, the clinical crowns of STL models in the T0, T1 and TX phases were intercepted, and the dentition integration models of different stages were obtained. The basal bone of the mandible in the T0 and T1 phases was reconstructed and saved in combination with the dentition integration model in the same period. In Geomagic control X64, the combined T0 model and the initial model were overlapped by the crown. The models in T0 and T1 phases were overlapped by “manual alignment+overall alignment” through the basal bone region of the mandible, and the accurate overlap of the dentition in the T0, T1 and TX phases was obtained.All the procedure was done by some same dentist.

***Measurement and calculation of tooth movement data:*** Three points, the midpoint of the line connecting the buccal cusp of the mandibular first premolar before treatment and the bilateral midpoints of the line connecting the proximal mesiobuccal cusps of the mandibular first molar, were selected to construct the datum plane. By connecting the midpoint of the line connecting the buccal cusp of the mandibular first premolar and the midpoints of the line connecting the proximal mesiobuccal cusps of the mandibular first molar, the datum plane was turned 90° through the lines to form a sagittal plane. The coronal plane was obtained by orthogonal construction from the datum plane, sagittal plane and the midpoint of the line connecting the mesiobuccal cusp of the mandibular first molar.

### Statistical analysis:

All data in this study were statistically analyzed using SPSS 20.0, and the measurement data conforming to the normal distribution was tested by T test, otherwise, the rank sum test was used, which was expressed as mean ± standard deviation (*x̅*±S). The confidence interval is 95%, p<0.05 was considered a statistically significant difference.

## RESULTS

In Group-A, the expected variations in the proximal and distal mesial direction of the crown and root of the mandibular first and second molars were not completely achieved, with statistically significant differences (p<0.05). Specifically, the crown was tilted by 2.97° and 3.07° in the distal direction, and moved by 0.88 mm and 1.58 mm in the buccal direction, with statistically significant differences (p<0.05) (Tables-I and II). The crown of the mandibular central incisor was labially moved by 0.61 mm and the root tip elongated by 0.35 mm, with statistically significant differences (p<0.05) (Table-III).

**Table-I:** Comparison of expected and actual variations of mandibular first molars in Group-A.

Measurement item	Actual variation	Expected variation	D-value	T value	P value
Crown axis inclination (°)	-2.52±2.05	0.45±1.78	-2.97±1.85	-5.075	0.001**
Crown proximal and distal distance (mm)	1.19±0.43	2.20±0.75	-1.01±0.43	-7.422	<0.001**
Crown buccolingual diameter (mm)	1.04±0.25	0.16±0.75	0.88±0.67	4.152	0.002*
Crown vertical height (mm)	-0.03±0.42	0.04±0.34	-0.08±0.51	-0.482	0.641
Root tip proximal and distal distance (mm)	0.48±0.44	2.22±0.71	-1.74±0.84	-6.566	<0.001*
Root tip buccolingual diameter (mm)	-0.10±0.54	-0.38±1.45	0.28±1.18	0.752	0.471
Root tip vertical height (mm)	-0.37±0.41	-0.21±0.75	-0.16±0.63	-0.806	0.441

* Means p<0.05,

** means p<0.01.

**Table-II:** Comparison of expected and actual variations of mandibular second molars in Group-A.

Measurement item	Actual variation	Expected variation	D-value	t/Z value	P value
Crown axis inclination (°)	-3.41±2.28	-0.34±2.41	-3.07±2.47	-3.933	0.003**
Crown proximal and distal distance (mm)	1.28±0.48	2.20±0.73	-0.92±0.32	-9.152	<0.001**
Crown buccolingual diameter (mm)	1.27(0.98,1.48)	-0.02(-0.55,0.13)	1.58(1.00,1.87)	-2.701*	0.007**
Crown vertical height (mm)	-0.13±0.16	0.02±0.83	-0.15±0.74	-0.659	0.526
Root tip proximal and distal distance (mm)	0.36±0.59	2.33±0.80	-1.97±0.94	-6.646	<0.001**
Root tip buccolingual diameter (mm)	-0.25(-0.50,0.34)	-0.07(-0.36,1.06)	-0.02(-0.30,0.21)	-0.561*	0.575
Root tip vertical height (mm)	-0.55±0.58	-0.16±0.96	-0.40±1.01	-1.246	0.244

* Means p<0.05,

** means p<0.01.

**Table-III:** Comparison of expected and actual variations of mandibular central incisors in Group-A

Measurement item	Actual variation	Expected variation	t value	P value
Crown axis inclination (°)	1.33±2.62	0	1.603	0.143
Crown proximal and distal distance (mm)	-0.06±0.31	0	-0.645	0.535
Crown buccolingual diameter (mm)	0.61±0.67	0	2.863	0.019*
Crown vertical height (mm)	0.19±0.36	0	1.611	0.142
Root tip proximal and distal distance (mm)	0.20±0.30	0	2.085	0.067
Root tip buccolingual diameter (mm)	0.20±0.72	0	0.864	0.410
Root tip vertical height (mm)	0.35±0.21	0	5.101	0.001*

* Means p<0.05, **means p<0.01.

Compared with the expected distalization, the actual distalization of mandibular first and second molars in Group-B showed that the crown was tilted 4.89° and 5.21°, with statistically significant differences (p<0.05). The crown and root of the mandibular second molar were depressed 1.56mm and 1.75mm, with statistically significant differences (p<0.05) (Tables IV and V). Moreover, the unexpected distalization of mandibular central incisors was not statistically significant (p>0.05) (Table-VI).

**Table-IV:** Comparison of expected and actual variations of mandibular first molars in Group-B

Measurement item	Actual variation	Expected variation	D value	t/Z value	P value
Crown axis inclination (°)	-3.61±1.42	1.28±0.91	-4.89±2.03	-6.371	0.001**
Crown proximal and distal distance (mm)	1.66±0.60	2.47±0.89	-0.81±0.48	-4.422	0.004**
Crown buccolingual diameter (mm)	1.80±0.67	1.40±0.62	0.40±0.66	1.620	0.156
Crown vertical height (mm)	-0.38±0.36	0.08±0.59	-0.46±0.62	-1.969	0.096
Root tip proximal and distal distance (mm)	0.74±0.54	2.76±0.79	-2.0l±0.96	-5.569	0.001**
Root tip buccolingual diameter (mm)	-0.10(-0.96,1.37)	-0.44(-2.07,0.07)	0.43(0.21,0.77)	-2.1974	0.028*
Root tip vertical height (mm)	-0.54±1.18	-0.27±0.77	-0.27±0.71	-1.027	0.344

* Means p<0.05,

** means p<0.01.

**Table-V:** Comparison of expected and actual variations of mandibular second molars in Group-B

Measurement item	Actual variation	Expected variation	D value	t/Z value	P value
Crown axis inclination (°)	-4.13±1.33	1.08±1.03	-5.21±2.04	-6.754	0.001**
Crown proximal and distal distance (mm)	2.16±0.42	3.02±0.32	-0.86±0.18	-12.424	<0.001**
Crown buccolingual diameter (mm)	2.30±0.70	1.61±0.60	0.69±0.84	2.167	0.073
Crown vertical height (mm)	-1.38±0.70	0.18±0.84	-1.56±0.90	-4.574	0.004**
Root tip proximal and distal distance (mm)	0.99±0.49	3.01±0.87	-2.02±0.72	-7.383	0.001**
Root tip buccolingual diameter (mm)	0.74±1.73	1.74±0.94	-1.00±1.49	-1.777	0.126
Root tip vertical height (mm)	-1.57±0.68	0.17±0.73	-1.75±0.67	-6.915	<0.001**

*Means p<0.05,

** means p<0.01.

**Table-VI:** Comparison of expected and actual variations of mandibular central incisors in Group-B.

Measurement item	Actual variation	Expected variation	t value	P value
Crown axis inclination (°)	-0.93±1.67	0	-1.471	0.192
Crown proximal and distal distance (mm)	0.03±0.28	0	0.262	0.802
Crown buccolingual diameter (mm)	-0.14±0.48	0	-0.776	0.467
Crown vertical height (mm)	0.03±0.31	0	0.246	0.814
Root tip proximal and distal distance (mm)	-0.4l±0.56	0	-1.951	0.099
Root tip buccolingual diameter (mm)	0.22±0.49	0	1.203	0.274
Root tip vertical height (mm)	0.38±0.52	0	-1.471	0.192

The mean distalization efficiency of the crown and root of mandibular first and second molars was 54.98% and 56.59%, while that in Group-B was 68.66% and 71.02%, with statistically significant differences (p<0.05) (Table-VII). The mandibular central incisors in Group-B showed reduced distalization in the coronal-labial direction and root tip distalization in the proximal-medial direction compared to Group-A, with statistically significant differences (p<0.05) (Table-VIII).

**Table-VII:** Comparison of the distalization efficiency of mandibular molars between the two groups.

Dental position	Distalization efficiency of Group-A (%)	Distalization efficiency of Group-B (%)	t value	P value
Mandibular first molar (crown)	54.98±8.87	68.66±11.16	-2.818	0.013*
Mandibular second molar (crown)	56.59±9.42	71.02±7.61	-3.349	0.004*
Mandibular first molar (root tip)	24.63±23.07	28.87±20.51	-0.390	0.702
Mandibular second molar (root tip)	16.17±21.04	33.45±17.53	-1.778	0.096
Mandibular second molar (crown)	56.59±9.42	71.02±7.61	-3.349	0.004*
Mandibular first molar (root tip)	24.63±23.07	28.87±20.51	-0.390	0.702
Mandibular second molar (root tip)	16.17±21.04	33.45±17.53	-1.778	0.096

* Means p<0.05, **means p<0.01.

**Table-VIII:** Comparison of the distalization efficiency of mandibular molars between the two groups.

Dental position	Distalization efficiency of Group-A (%)	Distalization efficiency of Group-B (%)	t value	P value
Mandibular first molar (crown)	54.98±8.87	68.66±11.16	-2.818	0.013*
Mandibular second molar (crown)	56.59±9.42	71.02±7.61	-3.349	0.004*
Mandibular first molar (root tip)	24.63±23.07	28.87±20.51	-0.390	0.702
Mandibular second molar (root tip)	16.17±21.04	33.45±17.53	-1.778	0.096

* Means p<0.05, **measns p<0.01.

## DISCUSSION

In this study, by combining CBCT with digital models, the characteristics of mandibular molar distalization with a clear aligner were analyzed, and the effects of micro-implant anchorage on molar distalization efficiency and anterior teeth anchorage protection were compared. In recent years, with the demand for more comfortable and aesthetic appliances, leading to the development of clear aligner therapy.^8^,^9^ Clear aligner therapy often requires the use of auxiliaries to improve the efficacy of orthodontic movement.^10^,^11^ Recent study showed that attachments mostly increase the effectiveness of orthodontic treatment with clear aligners, improving anterior root torque, rotation, and mesio-distal movement.^12^ In the No-micro implant anchorage group, all the crown roots of mandibular molars could not achieve the expected distal displacement, which is consistent with the research results of Rossini.^13^ Micro-implant anchorage can effectively improve the distal displacement efficiency of the mandibular molar crown, but as an “absolute anchorage”, it still cannot fully realize the expected distal displacement of the molar, which needs further verification.^14^ The mandibular molars moved in three dimensions. When sagittally upward, the crown inclination of the mandibular molars in both groups showed far-middle inclination compared with the expected change; when vertically upward, the mandibular molars in both groups showed exceeding expected depression, which is consistent with the vitro research result of Simon et al.,^15^ suggesting that the transparent aligner pushing molar distal displacement may also be suitable for cases with high angle tendency; when horizontally upward, the buccal movement of the molar crown was beyond expected in both groups, consistent with researches of Hong et al.^16^, which speculated that the buccal movement of molars was related to the shape of the dental arch, the deformation of the aligner and the direction of force application.^17^,^18^ In terms of the protective effect of mandibular central incisors anchorage, micro-implant anchorage can reduce the mesial inclination trend of incisors. In the No-micro-TADs group, mandibular central incisor elongated, which is consistent with the research results of Hahn et al.^19^

In the process of mandibular molar distalization assisted by micro-implant anchorage combined with a clear aligner, better protects the anchorage of the mandibular central incisor and improves the efficiency of the molar crown distalization.

### Limitations of this study:

The sample size was small, with limited clinical data available and limited persuasive conclusions. Further intervention trials are needed in the future to confirm these results.

## CONCLUSION

Orthodontists should comprehensively evaluate the molar distalization space and the three-dimensional position relationship between the lower anterior teeth and alveolar bone by CBCT before treatment. In addition, a reasonable anchorage type should be selected according to the actual situation of patients, the unexpected movement of teeth should be predicted, and the changes in the position of the anterior crown root should be closely monitored during treatment.

### Authors’ Contributions:

**GL** and **MC:** Carried out the studies, participated in collecting data, drafted the manuscript, are responsible and accountable for the accuracy and integrity of the work.

**NG:** Performed the statistical analysis and participated in its design.

**XS:** Participated in acquisition, analysis, or interpretation of data and draft the manuscript. All authors read and approved the final manuscript.
